# Glix 13, a New Drug Acting on Glutamatergic Pathways in Children and Animal Models of Autism Spectrum Disorders

**DOI:** 10.1155/2014/234295

**Published:** 2014-01-30

**Authors:** Annamaria Chiara Santini, Giovanna Maria Pierantoni, Raffaele Gerlini, Rosamaria Iorio, Yinka Olabinjo, Alfonso Giovane, Marina Di Domenico, Carla Sogos

**Affiliations:** ^1^Dipartimento di Pediatria e Neuropsichiatria Infantile, Università di Roma La Sapienza, Via dei Sabelli 108, 00185 Roma, Italy; ^2^Dipartimento di Medicina Molecolare e Biotecnologie Mediche, Università di Napoli Federico II, Via Pansini 5, 80131 Napoli, Italy; ^3^Dipartimento di Biochimica, Biofisica e Patologia Generale, Seconda Università degli Studi di Napoli, Via De Crecchio 7, 80138 Napoli, Italy; ^4^Dipartimento di Medicina, Chirurgia e Neuroscienze, Università di Siena, Policlinico Santa Maria alle Scotte, Viale Mario Bracci 16, 53100 Siena, Italy

## Abstract

Recently standardized diagnostic instruments have been developed in diagnostic and therapeutic procedures for Autism Spectrumv Disorders (ASD). According to the DSM-5 criteria, individuals with ASD must show symptoms from early childhood. These symptoms are communication deficits and restricted, repetitive patterns of behaviour. It was recently described by Bioinformatic analysis that 99 modified genes were associated with human autism. Gene expression patterns in the low-line animals show significant enrichment in autism-associated genes and the NMDA receptor gene family was identified among these. Using ultrasonic vocalizations, it was demonstrated that genetic variation has a direct impact on the expression of social interactions. It has been proposed that specific alleles interact with a social reward process in the adolescent mouse modifying their social interaction and their approach toward each other. In this review we report that the monoclonal antibody-derived tetrapeptide GLYX-13 was found to act as an N-methyl-D-aspartate receptor modulator and possesses the ability to readily cross the blood brain barrier. Treatment with the NMDAR glycine site partial agonist GLYX-13 rescued the deficit in the animal model. Thus, the NMDA receptor has been shown to play a functional role in autism, and GLYX-13 shows promise for the treatment of autism in autistic children.

## 1. Introduction 

Autism Spectrum Disorders (ASD) are diseases driven by abnormalities in reciprocal social interaction (SI) and by the limited and repetitive behaviours (American Psychiatric Association, 1994). In developing infants, the evolution of social behaviours and the ability to share affect with other people have been previously described [1]. Meltzoff showed that the interactions between infants and their caregivers suggest the child's ability to respond to the emotions of those around him [2, 3]. Kanner said that children with autism were “like in a shell, happiest when left alone, acted as if people were'nt [sic] there and failed to develop the usual amount of social awareness” [4]. What is really frustrating, for the reliability of behavioural diagnosis, is that for autism a specific biomarker has not yet been identified. It is well known that a central network in the pathology of psychiatric disorders is represented by glutamatergic signalling, through N-methyl-D-aspartic acid (NMDA) receptor [5]. This receptor is activated by glutamate when specific D-serine or glycine coagonists occupy its allosteric site [6]. Glycine is considered the main coagonist in the spinal cord and in the hindbrain, and it has a high affinity for extrasynaptic NMDARs. D-serine is the main coagonist in the forebrain [7], and it is characterized by a high affinity for synaptic NMDARs. In several brain regions (hippocampus, thalamus, and neocortex), NMDA receptor glycine/D-serine site is normally not saturated [8, 9] and, in this light, it was shown that the treatments of coagonists produce a modulation of affective behaviours in animal models [10].

Several mutations or allelic variants, that can influence brain development and behaviour, have been found in autism. Animal models have been employed to underline psychobiological determinants and early epigenetic influence [11]. In particular, juvenile mice are a popular species for genetic research [10] as well as in a more naturalistic context [12]. It was recently described that Bioinformatic analyses of 99 modified genes were associated with human autism. Gene expression patterns in the low-line animals show significant enrichment in autism-associated genes and the NMDA receptor gene family was identified among these. Recently, the monoclonal antibody-derived tetrapeptide GLYX-13 was found to act as an N-methyl-D-aspartate receptor modulator and it possesses the ability to readily cross the blood brain barrier. Treatment with the NMDAR glycine site partial agonist GLYX-13 rescued the deficit in the animal model. Thus, the NMDA receptor has been shown to play a functional role in autism, and GLYX-13 shows promise for the treatment of autism in autistic children.

## 2. GLYX 13, DMG, and NMDA Signaling

N-methyl-D-aspartate glutamate receptors (NMDARs) are involved in Ca^2+^ influx into neurons and are important to synaptic plasticity. When these receptors are uncontrolled they may trigger events that cause neuronal degeneration and death. This receptor-ionophore complex is functionally involved in modulating normal synaptic transmission, synaptic plasticity, and excitotoxicity in the central nervous system. Unlike other ligand-gated ion channels, the N-methyl-D-aspartate receptor-ionophore complex has a unique feature since it requires two distinct recognition sites by glutamate and glycine for its activation. Although the stoichiometry of native NMDARs is still uncertain, recombinant NMDARs appear to consist of at least one NR1 and one NR2 subunits, and most evidence now suggests that NMDARs assemble as tetramers containing 2 NR1 and 2 NR2 subunits. Many glutamate receptors have been found in the central nervous system but the glycine site of NMDA receptor possesses the distinctive ability to be strychnine-insensitive. Because of this specificity and the number of clinically relevant functions in which the NMDA receptors are involved, its glycine site is a potentially important target for drug development. Therefore, a noticeable increase occurred in the exploitation of pharmacological agents that interact selectively with the glycine binding site. For example, glycine and D-serine were found to be helpful in reducing some of the negative symptoms of schizophrenia [13] when used to augment antipsychotic therapeutics. The partial agonist, D-cycloserine (DCS), has been shown to have cognitive-enhancing properties in vivo. However, these compounds show desensitization after chronic administration. An increasing number of glycine site modulators have been described as appearing to have therapeutical potential. In 1991 was produced a monoclonal antibody, B6B21, that significantly elevates long-term potentiation when applied to CA1 pyramidal cell apical dendrites in rat hippocampus [14]. This antibody was found to bind at strychnine-insensitive glycine sites thus demonstrating its direct binding to N-methyl-D-aspartate receptors. In 2005, the B6B21 was transformed into a family of small peptides called Glyxins [15]. The GLYX-13, a tetrapeptide (TPPT-amide) originating from that antibody, was found to act as a NMDA receptor modulator similar to the partial agonist D-cycloserine and potentiates learning when administered intravenously to rats undergoing hippocampus-dependent trace eyeblink conditioning. Pharmacological and electrophysiological experiments demonstrate that GLYX-13 modulates the NMDA receptor in a glycine-like manner as a partial agonist. On the other hand, it is still unclear if GLYX-13 precisely mimics the action of the monoclonal antibody B6B21 from which it is derived. It is likely that GLYX-13 acts on a different site that indirectly affects glycine binding. Data obtained from behavioral studies suggest that systemic administration of peptides acting as NMDA receptor modulators can facilitate hippocampus-dependent learning. GLYX-13 administration in rats showed enhancements of hippocampus-dependent trace eyeblink conditioning that were similar to those obtained with the antibody B6B21 and with D-cycloserine, the partial agonist of the glycine site on the NMDA receptor. It is worthy to note that chronic administration of DCS can lead to desensitization and the monoclonal antibody B6B21 does not cross the blood brain barrier; instead GLYX-13 easily crosses the blood brain barrier making it a good candidate for clinical use as a cognitive enhancer.

## 3. Genetic Variation Influence in Autistic Animal Models

Knockouts in gene-targeted mice have been produced. They carry mutations in genes that have been associated with a SDA [16]. Genetic correlations include allelic variants of fragile-X [17]. Allelic variants have been found to be associated with ASD and gene-targeted mice can be generated to study development of receptor mechanisms in order to evaluate potential therapeutic strategy. Other evidences have been shown in fragile X mouse models, and several other strains, most notably the BTBR mouse, underline the importance of animal model in autism research [18]. Several evidences show a genetic predisposition to autism that involves a reduced expression of gene coding for Hepatocyte growth factor receptor (HGFR), also known as mesenchymal-epithelial transition factor (MET). In particular a 2-fold decrease in MET promoter activity and altered binding of specific transcription factor was observed in 204 autism families [19]. A critical step is to continue to push for more nuanced measures of mouse socialability, including the capacity to express emotion and to respond to emotional expressions of others. Several studies [20, 21] demonstrated that prepubescent mice from the B6 strain are particularly prosocial, whereas age-matched BALB mice are less reactive to social ability. Interestingly, adult mice from these two genetic backgrounds appear to be much less distinct than juvenile mice in terms of their SI [22, 23].

According to Panksepp et al. [24], genetic variation has a direct impact on the expression of SI which can be divided into the following: (i) sniffing or snout contact with the head/neck/mouth area, (ii) sniffing or snout contact with the flank area, (iii) direct contact with the anogenital area, (iv) social pursuit within one body-length as the stimulus mouse moved continuously throughout the cage, and (v) social grooming. SI were evaluated by ultrasonic vocalizations and related underlying reward. The authors also proposed that specific alleles are involved in a social reward process in the adolescent mouse, modifying SI and to approach each other.

## 4. GLYX-13 Treatment in Animal Models

Adolescent as well as young mammals exhibit a characteristic form of SI known as social play behaviour or rough-and-tumble play. This form of social behaviour is considered a fundamental step for the development of social and cognitive skills. Hence, in order to study disorders of neural development, young rats are generally used as animal model. In fact, young rats exhibit the highest rates of social rough-and-tumble play behavior from among all species tested [25]. During testing, animals are videotaped and high frequency ultrasonic vocalizations were recorded. In fact, several researches on rat brain have shown a correlation between high frequency ultrasonic vocalizations (USVs) and anticipatory affective states. More precisely, long low-frequency (approximately 22 kHz) USVs occur during anticipation of punishment or avoidance behavior, whereas short high frequency (approximately 50 kHz) USVs typically occur during anticipation of reward or approach behavior. Thus, long 22 kHz USVs may be an indicator of negative activation state, whereas short 50 kHz USVs may instead indicate a state of positive activation [26]. Thus, USVs recording can be used to assay the rat emotional state as consequence of drug administration. This type of animal model was used to study the effect of GLYX 13 in autism. An animal model that displays analogous symptoms of autism was created using rats that show low rates of prosocial ultrasonic vocalizations (i.e., frequency modulated 50 kHz USVs) in response to rough-and-tumble play behavior. According to Moskal the low-line animals used for the experimental study found that lower rates of play-induced prosocial ultrasonic vocalizations correlate to an increased proportion of monotonous ultrasonic vocalizations compared to randomly bred animals [27]. The low-line selection was also screened by microarray gene expression and significant gene changes in brain regions in low-line animals were found compared to nonselectively bred random-line animals. The administration of GLYX-13 at a dose of 50 mg/kg (s.c.) significantly increased rates of play-induced pro-social USVs and significantly decreased the proportion of total USVs that are monotonous (i.e., pure tone whistles without any detectible frequency modulation) [28].

## 5. A New Candidate Drug in Autistic Children

ASDs cover a heterogeneous group of neurodevelopmental disorders defined behaviourally by three core disturbances: marked deficits in interpersonal SI, disrupted verbal and nonverbal communication, and restricted repetitive and stereotyped patterns of behaviour and interests [29, 30]. The ASD phenotype includes the classical or typical autistic disorder (AD), Asperger syndrome (AS) characterized by no general delay in language or cognitive development, and pervasive developmental disorders not otherwise specified (PDD-NOS), which is a milder condition that includes some, but not all, of the symptoms associated with classic autism. Once considered a rare clinical entity, autism is now considered common, with the most recent prevalence estimate being around 1 in 150 [31, 32]. In the scientific literature, there is varying support for a wide spectrum of hypotheses regarding the causes of autism: from studies showing that genes play a greater role in the risk for autism than in any other common neuropsychiatric disorder [33] to studies implicating disruptive environmental factors during neurodevelopment in genetically susceptible individuals [34]. Nevertheless, it is becoming increasingly obvious that a single cause or unifying theory is unlikely to account for what is now better referred to as “the autisms” [35, 36]. According to DSM-V, autism spectrum disorders (ASDs) can be diagnosed in a patient in early childhood with persistent deficits in social communication and SI. Autism is also manifested as deficits in social emotional reciprocity, deficits in nonverbal communicative behaviours, and deficits in developing and maintaining relationships. The patient has to be characterized by restricted, repetitive patterns of behaviour, interests, or activities. A paper aimed to compare the joint engagement of children with autism, children with Down syndrome, and their typically developing peers indicated that autism and Down syndrome often affect a young child's joint engagement experiences during social interactions with a caregiver [37]. Children with autism rarely coordinated attention to a shared object as normal developing peers do. In contrast, children in the Down syndrome group readily shared events with their partners but they were less likely to attend to symbols during these periods.

No specific biomarker for autism has been identified yet in order to improve the reliability of behavioural diagnosis. It was shown that the GABA system is related to pathophysiology of autism [38]. The exact pathophysiology of autism is yet unknown but the N-methyl-D-aspartate receptor (NMDAR) has received great attention as a possibility of treatment. In order to study its implications in this disorder, animal models have been used. A rat model has been developed for autism features such as social and communication deficits and repetitive and restrictive behaviours. Evidences previously described include dysfunction of NMDARs; as is known genetic risk factors suggest that its hypofunction is involved in pathogenesis. The NMDAR pathway could become a target for the development of a variety of therapeutic drugs as glycine site modulators. In fact, it is involved in learning and memory formation and also in a number of neuropathologies [39]. NMDAR role is validated by the effects obtained upon D-cycloserine treatment. D-cycloserine is an antibiotic and a partial agonist at NMDA; its significant improvement in social withdrawal has been shown and it has been proposed as a treatment for autism by several authors. Posey et al. described the effects of DCS on the Autism Spectrum Disorder symptoms [40]. The therapeutic program consisted of a single-blind placebo lead-in phase about the treatment of 10 drug-free subjects (5–27 years old) with autistic disorder. Three different doses of D-cycloserine were used and patients followed for two weeks. Measures used for subject ratings included the Clinical Global Impression (CGI) scale and Aberrant Behavior Checklist. On the highest dose, subjects enrolled for this therapeutic approach had statistically significant improvement in social withdrawal. Adverse effects reported included motor tics and increased echolalia in two subjects. Priestley et al. [41] suggested that partial agonists at glycine site may be better therapeutic candidates: acting as weak agonists, they would facilitate receptor activation without creating the risk of overactivating the receptors and acting as antagonists, they would allow normal synaptic transmission to take place while simultaneously suppressing receptor hyperactivity, through the NMDA signaling.

## 6. Concluding Remarks

GLYX 13 belongs to a new class of NMDAR glycine site modulators with therapeutic potential and could have a clinical value. Several studies have reported that important peptides derived from monoclonal antibody have been created for therapeutic approach. In order to provide useful novel therapeutics in autism, the mimetic peptide GLYX-13 has been produced and is added to the list of CDR-derived functional peptides (complementary determining regions). Recent studies show that GLYX-13 readily crosses the blood brain barrier and modulates the NMDA receptor in a glycine-like way decreasing the deficit in animal models and giving hope for the treatment of autism in children. Burgdorf et al. [42] showed that this drug can be used in association with ketamine and produced antidepressant-like effect. These treatments are currently in a Phase II clinical development program for treatment-resistant depression. Several reports explain how NMDA receptors are involved in neuropsychiatric disorder, as well as development of symptoms for schizophrenia. However, open questions concerning the molecular mechanism of NMDAR dysfunction yet remain.
